# Importance of Crosstalk Between Chronic Lymphocytic Leukemia Cells and the Stromal Microenvironment: Direct Contact, Soluble Factors, and Extracellular Vesicles

**DOI:** 10.3389/fonc.2020.01422

**Published:** 2020-08-19

**Authors:** Nathan Dubois, Emerence Crompot, Nathalie Meuleman, Dominique Bron, Laurence Lagneaux, Basile Stamatopoulos

**Affiliations:** ^1^Laboratory of Clinical Cell Therapy, ULB-Research Cancer Center (U-CRC), Jules Bordet Institute, Université Libre de Bruxelles (ULB), Brussels, Belgium; ^2^Department of Hematology, Jules Bordet Institute, Brussels, Belgium

**Keywords:** chronic lymphocytic leukemia, microenvironment, mesenchymal stromal cells, extracellular vesicles, prognostic factor

## Abstract

Chronic lymphocytic leukemia (CLL) is caused by the accumulation of malignant B cells due to a defect in apoptosis and the presence of small population of proliferating cells principally in the lymph nodes. The abnormal survival of CLL B cells is explained by a plethora of supportive stimuli produced by the surrounding cells of the microenvironment, including follicular dendritic cells (FDCs), and mesenchymal stromal cells (MSCs). This crosstalk between malignant cells and normal cells can take place directly by cell-to-cell contact (assisted by adhesion molecules such as VLA-4 or CD100), indirectly by soluble factors (chemokines such as CXCL12, CXCL13, or CCL2) interacting with their receptors or by the exchange of material (protein, microRNAs or long non-coding RNAs) via extracellular vesicles. These different communication methods lead to different activation pathways (including BCR and NFκB pathways), gene expression modifications (chemokines, antiapoptotic protein increase, prognostic biomarkers), chemotaxis, homing in lymphoid tissues and survival of leukemic cells. In addition, these interactions are bidirectional, and CLL cells can manipulate the normal surrounding stromal cells in different ways to establish a supportive microenvironment. Here, we review this complex crosstalk between CLL cells and stromal cells, focusing on the different types of interactions, activated pathways, treatment strategies to disrupt this bidirectional communication, and the prognostic impact of these induced modifications.

## Introduction

During the last 20 years, the number of reports dealing with the interaction between chronic lymphocytic leukemia (CLL) and the surrounding cells constituting its microenvironment has increased exponentially. Focus has been placed on one particular actor in the bone marrow microenvironment that is also present in several lymphoid tissues: mesenchymal stromal cells (MSCs). The first evidence that the microenvironment is crucial for leukemic cell survival comes from CLL cells undergoing rapid apoptosis when cultured alone but are rescued when cultured in direct contact with stroma. In the nineties, Panayitidis et al. (1) and our group (2) were among the first to highlight that this dependency plays a critical role in the pathophysiology of CLL. In addition to direct contact via adhesion molecules, CLL cells, as well as MSCs, communicate via the secretion of soluble factors (including chemokines, cytokines, and growth factors) that influence leukemic cell trafficking and homing within bone marrow niches. These niches are sanctuaries providing different survival signals and protect CLL cells from spontaneous and drug-induced apoptosis. Parallelly, in secondary lymphoid tissues such as lymph nodes, CLL cells interact with another actor of the stromal microenvironment: the follicular dendritic cell (FDC). FDCs are involved in the homing, the survival (3) and the proliferation (4) of CLL B cells within the germinal centers by producing several cytokines and chemokines (5, 6), by expressing several adhesion molecules (7–9) for CLL cells or by direct cell contact (3, 10). Recently, a new communication method via the production of extracellular vesicles (EVs) also adds complexity to these bidirectional CLL/MSC interactions. Using different interactions, the leukemic clone is able to manipulate the surrounding cells to recruit or to be recruited, re-educate and literally transform the surrounding cells into a protective microenvironment. In the present review, we summarize all the available data describing the crucial role of the stromal microenvironment in CLL, different manners of communication, activated pathways and how they can be targeted and, finally, the impact of this crosstalk on CLL patient prognosis.

## Chronic Lymphocytic Leukemia

Chronic lymphocytic leukemia (CLL) is one of the most common leukemias in Western countries, accounting for 37% of cases with an incidence of 4.9 per 100 000, a male/female ratio of 1.9, a median age at diagnosis of 70 years old and more than 20 000 new cases diagnosed in 2019 in the United States (11). The disease is caused by the clonal expansion of leukemic B cells and is characterized by the expression of CD5, CD19, and CD23 (12), a defect in apoptosis (13, 14), and for the majority of CLL cells, a resting state (15, 16). However, proliferation centers have been described (15, 17) and using a deuterium oxide *in vivo* labeling method in which patients consumed deuterated water (^2^H_2_O), the lymph node has been identified as the anatomical site harboring the largest fraction of newly born cells with a calculated birth rate up to 3.3% of the clone per day (18). Another characteristic of CLL is its clinical heterogeneity (19). Some patients have an indolent course and live decades without any treatment, while others have a more aggressive disease, need early treatment and have a shortened survival. This heterogeneity can be predicted by a plethora of prognostic markers. The mutation status of the immunoglobulin heavy chain region (IgHV) (20), some cytogenetic abnormalities based on the Döhner classification [del(17p), del(11q), trisomy 12, del(13q)] (21), the expression of zeta–associated protein 70 (ZAP70) (22), lipoprotein lipase (LPL) (23), CD38 (24), CD49d (25), CD69 (26), some microRNAs [miR-29c and miR-223 (27), miR-34a (28), miR-150 (29)], and the presence of point mutations (tumor protein 53–TP53) (30).

While the cell origin of the disease is still under debate, the scientific community agrees that B cell receptor (BCR) pathways are crucial for the selection, development, proliferation, and survival of CLL clones (31–33). The BCR is composed of a surface immunoglobulin (Ig) made of 2 heavy and 2 light chains that are non-covalently associated with the heterodimer Ig-α/Ig-β (also known as CD79a/CD79b). External antigens from the microenvironment (34) as well as intra-BCR self-antigens (35) trigger BCR signaling, leading to the recruitment of tyrosine kinases [spleen tyrosine kinases (SYKs) and Lck/Yes novel tyrosine kinase (LYN)] that phosphorylate the immunoreceptor tyrosine-based activation motifs (ITAMs) of Ig-α/Ig-β (36). This induces a cascade of downstream events, including activation of Bruton's tyrosine kinase (BTK) (37), phosphoinositide 3-kinase (PI3K) (38), protein kinase C (PKC) and ras-dependent extracellular signal-regulated kinase (ERK) (39), that ultimately lead to the upregulation of nuclear factor kappa B (NFκB) (40). This signaling cascade promotes CLL B cell survival (41, 42) and has therefore been considered a very potent therapeutic target that we will discuss in this review (43, 44).

## Mesenchymal Stromal Cells

Mesenchymal stromal cells (MSCs) are among the first actors in the CLL microenvironment that have been studied, even if, at that time, they were not called MSCs (1). These cells were discovered in 1968 by Friedenstein et al., who were the first to report an adherent fibroblastic-like cell population that was able to differentiate into osteoblasts, chondrocytes or adipocytes (45). In 1991, these cells were named “mesenchymal stem cells” by Caplan et al. (46), and from then, the term “MSC” has been popular. The first source of MSCs was found in bone marrow, but several other sources have been described (adipose tissue, Wharton's jelly of the umbilical cord, dental pulp, skin, etc.) in numerous organs in which cell renewal is needed (47). MSCs are generally recovered by simple plastic adhesion, resulting in a heterogeneous cell population with different stemness potentials. Therefore, to avoid any controversies, the term “stem” in “mesenchymal stem cell” has been replaced by “stromal,” referring to a bulk population with secretory, immunomodulatory, and differentiation potential and homing properties (48). MSCs are heterogeneous cells and cannot be defined by a single marker. Therefore, in 2006, the International Society for Cellular Therapy (ISCT) proposed a set of minimal criteria to define human multipotent MSCs (49): [1] MSCs must adhere to plastic when maintained in culture; [2] MSCs should express (≥95%) CD105, CD73, and CD90, as measured by flow cytometry but should not express (≤ 2%) hematopoietic markers (CD45, CD34, CD14 or CD11b, CD79a or CD19, and HLA class II); and [3] finally, MSCs should be able to differentiate into osteoblasts, adipocytes and chondroblasts. The number of MSCs in bone marrow aspirate represents ~0.01–0.001% of the total population of nucleated cells; therefore, in the majority of cases, MSCs require *ex vivo* expansion to obtain a reasonable number of cells to establish a feeder layer. Although these *ex vivo* expanded MSCs could be different from native MSCs, some studies have already shown that MSCs obtained from CLL patients present different properties compared to healthy MSCs. In 1995, our group observed increased production of transforming growth factor β1 (TGFβ1) by CLL patient-derived stromal cells compared to that of healthy stroma (50, 51). Pontikoglou et al. demonstrated that CLL patient-derived MSCs exhibit a similar phenotype compared to healthy MSC, as well as a similar differentiation potential and a CLL apoptosis protection. On the other hand, they also showed that these cells have defective cellular growth due to increased apoptotic cell death and exhibit aberrant production of stromal cell derived factor 1 [SDF1, also named C-X-C motif chemokine ligand 12 (CXCL12)] or TGFβ1, two important cytokines that are crucial for the survival of leukemic cells (52). Janel et al. confirmed this low proliferative capacity (53). In addition, they also observed increased culture failure, a polygonal aspect and an increased proportion of senescence-associated β-galactosidase-positive cells in CLL patient-derived MSCs compared to those of healthy MSCs (53). These two *ex vivo* reports suggest that CLL patient derived MSCs are probably already dependent on leukemic clones, at least for their long-term survival, as the leukemic clones are dependent on MSCs for their own survival. While this phenomenon has been reported for other malignancies such as multiple myeloma (54), the dependency of MSCs from CLL cells and their low proliferation rate have never been directly demonstrated *in vivo*. In addition, it should be noted that several reported differences between CLL-MSCs and age-matched healthy individual MSCs are known as senescence-associated [reviewed in (55)] and are therefore not necessarily linked to the disease itself. This is an important limitation of *in vitro* studies with MSC: their *ex vivo* expansion does not allow the study of “native” MSCs under physiological situation.

## Follicular Dendritic Cells

**Figure 1:**
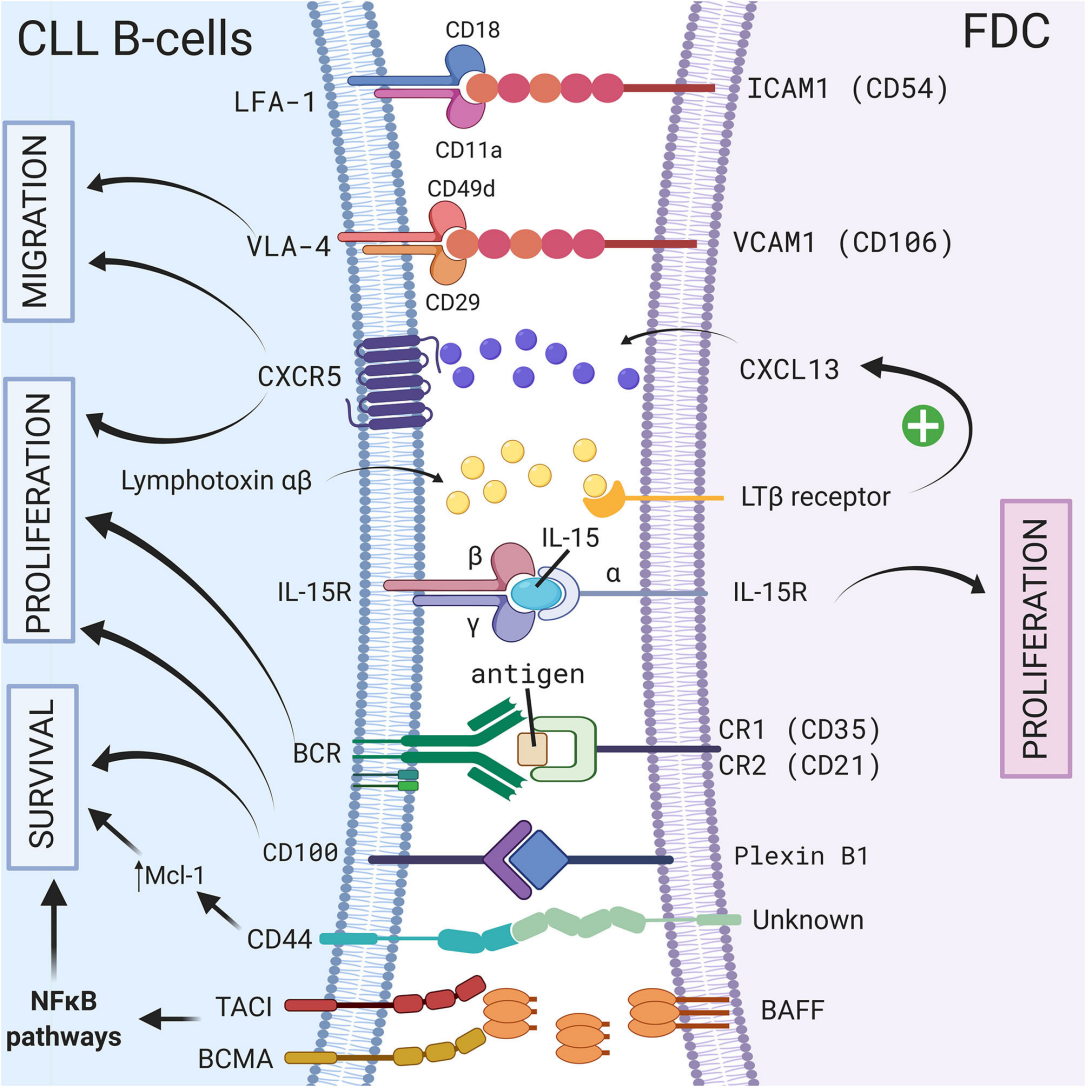
Crosstalk between CLL B-cells and FDCs via direct contact or soluble factors. In secondary lymphoid organs, CLL B cells interact with FDCs via different molecules, receptor/ligand couples including ICAM1/LFA-1 (8), VCAM1-VLA-4 (8), CD100/Plexin-B1 (9), immune complex/BCR (64), CXCR5/CXCL13 (10), BCMA/BAFF or TACI/BAFF (65), or by transpresentation of IL-15 from FDCs to germinal center B cells (6). These interactions could lead to a leukemic cell survival via a CD44-dependent mechanism involving up-regulation of Mcl-1 in CLL B cells (3), the activation NF-κB pathway (65), the migration, or the proliferation of leukemic cells. Figure created with BioRender.com.

Follicular dendritic cells (FDCs) are accessory cells located in the central region of primary follicles and in the light zone of normal germinal centers (56). Based on their dendritic appearance, FDCs were mistakenly considered as a subset of conventional dendritic cells. However, FDCs are from stromal origin and emerge from perivascular precursors (57), unlike conventional dendritic cells which are of hematopoietic origin. They also have different functions: one of the most important features of FDCs is their ability to capture antigen–antibody complexes (called “immune complexes”—IC) on their cellular surface through the involvement of complement receptors 1 (CR1 or CD35) and 2 (CR2 or CD21) (58), and present unprocessed antigen to the B cells. This was observed for the first time in 1965 using high-resolution electron microscopic autoradiographs and radioactively labeled microbial antigens (59). In the following years, several different names such as dendritic macrophages (60) or dendritic reticular (61) cells were used for these cells. In 1978, Chen et al. finally introduced the name FDCs (62) but admitted later that this name was not ideal. However, even if it was demonstrated that FDCs did not express class II MHC like conventional dendritic cells, the name FDCs still remains. Because of their ability to bind IC, FDCs are indispensable for secondary and tertiary lymphoid organ development and maintenance. FDCs are normally localized in secondary lymphoid organs such as the spleen or the lymph node (10) however, in CLL patients, FDCs have also been observed in nodular bone marrow infiltrates (63). Because of their cytokine secretion, the adhesion molecules they carry, their ability to activate BCR signaling, and their protective effect on the survival of CLL B cells, FDCs represent another important player of the stromal microenvironment, particularly in secondary lymphoid organs. A schematic representation of the different CLL/FDC interactions is shown in Figure 1 and is discussed below.

## Direct Contact: CLL/Stroma Coculture, Homing, and Adhesion Molecules

When CLL B cells are removed from the human body and plated alone in culture, they rapidly undergo apoptosis (13, 14). Co-culture of CLL B cells with MSCs prevents this cell death (1, 2). However, efficient protection is achieved when the two cell types are in contact, since separation with a filter prevents the protection from apoptosis (2, 66–69). In addition to protection from spontaneous apoptosis, this effect can be extended to drug-induced apoptosis (67, 70, 71). Since the first reports with primary MSCs, apoptosis protection has been confirmed with different stromal cell lines, such as HS5 (human) or M210B4 (mouse) (67). During co-culture, CLL B cells migrate spontaneously to and beneath the feeder layer of MSCs. This phenomenon is called pseudoemperipolesis to distinguish it from true emperipolesis, which involves penetration of living cells by other cells (66, 70, 71). This migration is induced by chemoattractants produced by MSCs, especially CXCL12, which will be discussed in the next chapter. Similarly, *in vitro* coculture of CLL B cells with HK cells, a follicular dendritic cell line, rescues CLL cells from spontaneous and drug induced apoptosis (3). Interestingly, this protection is at least partially mediated by a CD44-dependent mechanism involving up-regulation of Mcl-1 in CLL B cells (3).

The binding of CLL B cells to stromal cells requires simultaneous action of β1 integrin (ITGB1, also known as CD29) and β2 integrin (ITGB2, also known as CD18) (72, 73). The activation of these integrins occurs by heterodimerization, creating a conformational change, increased affinity and redistribution on the plasma membrane (74). These results were consistent with a more recent report that very late antigen-4 (VLA-4), which is composed of a CD49d and CD29 dimer, is an important integrin for retention of CLL cells in the microenvironment by interacting with its ligand vascular cell adhesion molecule 1 (VCAM-1 or CD106) on stromal cells (75). In addition, Brachtl et al. demonstrated a prominent role of CD49d in the homing of CLL cells to bone marrow niches and in human bone marrow infiltration (76). Interestingly, activation of VLA-4 and lymphocyte function-associated antigen 1 (LFA-1, which is composed of CD11a and CD18—also known as αLβ2 integrin) is triggered by CXCL12 produced by MSCs (74, 77), notably via the Janus kinase 2 (JAK2) pathway (78) but also by BCR stimulation, reinforcing the adhesive capacities of CLL B cells (79). While LFA-1 is involved in chemokine-mediated migration of CLL cells from patients with lymphadenopathies (80), in cells from the majority of CLL patients, there is a defect in chemokine-induced inside-out activation of LFA-1 (81). Interestingly, this defect can be overcome by the engagement of vascular endothelial growth factor (VEGF) receptor(s) and VLA-4 by their respective ligands (81). In addition to this activation defect, Hartmann et al. also showed that CLL cells expressed significantly reduced LFA-1 due to low β2 integrin transcripts compared to healthy B cells (77). For some authors, LFA-1 is not involved in the adhesion of CLL cells to bone marrow MSCs (73) but is important for their adhesion to FDCs that express high level of ICAM1 (7). Indeed, FDCs expressed ICAM1 and VCAM1 allowing, the interaction with LFA-1 and VLA-4, respectively, on CLL B cell surface (8). Granziero et al. also highlighted the interaction between CD100 (present on CLL B cell surface) and Plexin-B1 (present on bone marrow MSCs and FDCs) and showed that it extends CLL B cell viability and enhances proliferation (9).

Coculture of leukemic cells with MSCs is associated with actin polymerization (71, 82) and consequent cytoskeletal remodeling in CLL cells. For other leukemias such as chronic myeloid leukemia (83, 84) and acute lymphoblastic leukemia (85–87), tunneling nanotubes have been identified as a novel mode of intercellular crosstalk. Tunneling nanotubes are long and thin membranous structures that allow the exchange of material [such as mitochondria (85), vesicles or proteins (83)] between leukemic cells and stromal cells. While it has never been described in CLL, it is not impossible that tunneling nanotubes between CLL cells and MSCs could be a new way of communication allowing the transfer of mitochondria or proteins to CLL cells, as reported for other leukemias. However, we should be cautious when interpreting these results since the majority of data available today for CLL/microenvironment interactions represent a two-dimensional view of an *in vitro* coculture experiment. Therefore, new 3D culture systems have been proposed to study leukemia cells (88–90) and 3D coculture systems for studying specifically CLL/microenvironment interactions are under investigation, as presented in recent hematology congresses (91–93).

The contact between CLL B cells and MSCs induces dramatic gene expression modifications (94, 95), including an increase in antiapoptotic molecules such as B cell lymphoma 2 (BCL2) (96, 97), B cell lymphoma-extra large (BCL-XL) (96, 98), myeloid leukemia cell differentiation protein 1 (MCL1) (67, 71, 98), and β-catenin (95), as well as soluble factors that will be discussed below. Caveolin was also suggested to play a role in CLL survival in coculture: indeed, caveolin is increased in cocultures of CLL B cells with the NK. Tert stromal cell line (99) and play a role in CLL development in the Eμ-TCL1 mouse model (100). Numerous pathways are consequently activated within CLL B cells, including Toll-like receptor (94) and BCR (29). How BCR signaling is activated by MSCs is still unclear, but Binder et al. suggested that a BCR with a common stereotyped heavy chain complementarity-determining region 3 [from “subset1” (33)] recognizes vimentin and calreticulin, which are highly expressed on stromal cells (34). Interestingly, blocking vimentin by recombinant soluble CLL BCR reduces stromal-mediated apoptosis protection (34). In secondary lymphoid organ context, FDCs are able to stimulate BCR and activate normal B cells via FDC-bound antigen associated with CR1/2 (64). Heinig et al. identified follicular stromal networks that locally interact with leukemia cells isolated from Eμ-Tcl1, a CLL mouse model (10). In addition, these authors showed that the majority of leukemic cells in contact with FDCs expressed proliferation markers, suggesting that FDCs could participate to the stimulation and proliferation of CLL B cells in germinal centers (10).

Other authors highlight the possible epigenetic modifications induced by stromal cells. Xu et al. observed that CLL B cell protection in the presence of the murine stromal cell line HESS-5 is associated with hypomethylation of the trimethylation of lysine 27 on histone H3 protein subunit (H3K27me3) (101). Coculture with different stromal cell lines showed increased oxidative phosphorylation in CLL, which probably helps to increase their metabolism, allowing these cells to meet the energy demands for transcription and translation (102).

**Figure 2:**
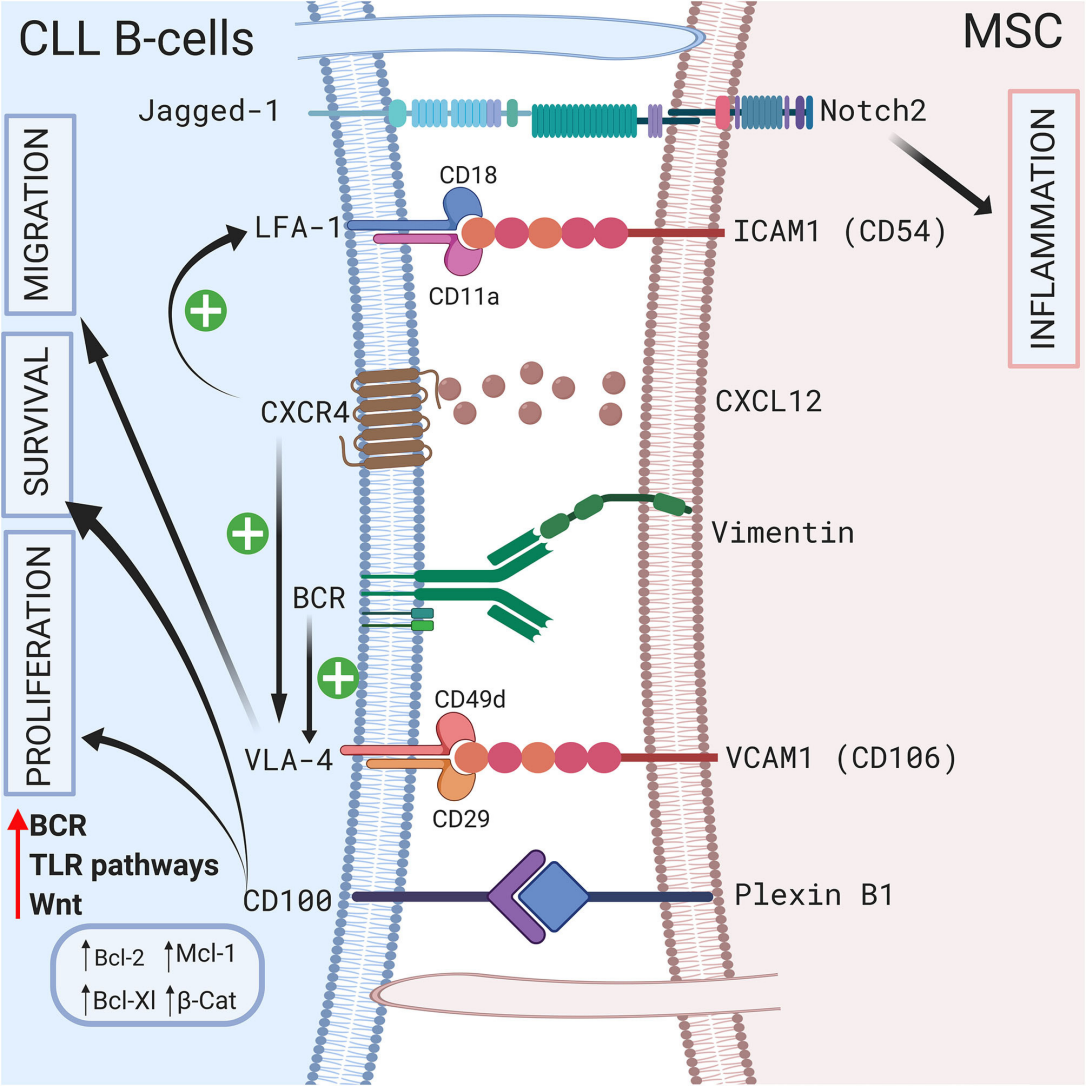
Crosstalk between CLL B-cells and MSCs via direct contact. CLL B-cells interact with MSCs by direct contact by different ways. First via pseudopods that increase cell surface contact. Second by different receptors and ligands including Jagged1/Notch2 (95), LFA-1/VCAM1 (72, 73), VLA4/VCAM1 (75), CD100/Plexin B1 (9), BCR/vimentin (34). Interestingly the CXCR4/CXCL12 axis plays a central role by triggering VLA-4 and LFA-1 axis (74, 77). BCR stimulation also increase VLA-4 (79). All these interactions lead to the proliferation, the migration and/or the survival of leukemic cells by inducing the upregulation of several anti-apoptotic protein including Bcl-2 (96), Mcl-1 (67, 71, 98), or Bcl-XL (96, 98). Figure created with BioRender.com.

Direct contact between CLL B cells and stromal cells also induces modifications in the stromal cells. Mangolini et al. showed that in a coculture system, neurogenic locus notch homolog protein 2 (Notch2) is activated in MSCs and regulates genes involved in inflammation and extracellular matrix formation, which are both important components of the CLL microenvironment (95). In addition, these authors demonstrated that coculture stabilizes β-catenin in CLL, activating the wingless integration site (Wnt) pathway. A schematic representation of the different cell-to-cell contact interactions is shown in Figure 2.

## Soluble Factors: Cytokines, Chemokines, and Growth Factors

Soluble factors play a key role in CLL B cell trafficking and homing (103). Leukemic cells could travel in the body from the peripheral blood to the bone marrow, where they receive survival signals. Numerous cytokines are produced by bone marrow MSCs, but one of the most studied cytokines is CXCL12, which interacts with its receptor, C-X-C motif chemokine receptor 4 (CXCR4, also known as CD184), on leukemic cells (66, 69, 75). Möhle et al. showed that CLL B cells overexpress CXCR4 compared to normal B cells, making them more able to respond to CXCL12 (104). This was confirmed by *in vivo* studies showing that higher CXCR4 levels increase the risk for lymphoid organ infiltration (105). Interestingly, CXCR4 expression is dynamically regulated on CLL cell surface. Using CXCR4 and CD5 staining on deuterium-labeled cells, Callissano et al. indeed showed that CXCR4^dim^CD5^bright^ cell fraction is enriched in young, vital and proliferating cells while CXCR4^bright^CD5^dim^ fraction is composed of older, less robust and resting cells (105). Based on these data, these authors hypothesized lifecycle of CLL B cells: on a stroma, CLL cells could be stimulated and activated. Then, they begin to divide, to upregulate CD5 and to downregulate CXCR4, detaching from the stroma, and are released in the circulation (CXCR4^dim^CD5^bright^ phenotype). Over time, cells begin to re-express CXCR4 (CXCR4^bright^CD5^dim^ phenotype). These cells have the greatest chance of detecting and following a CXCL12 gradient, thereby reentering lymphoid solid tissue and receiving prosurvival stimuli. The binding of CXCL12 to CXCR4 induces actin polymerization, cytoskeletal remodeling, transendothelial migration and tissue homing of leukemic cells (66). In addition to its chemoattractant effects, CXCL12 has also been shown to be a survival factor (71, 106). Following CXCL12 binding, CXCR4 is downregulated on the CLL B cell surface by endocytosis, making the cells less responsive to CXCL12 and allowing their recirculation in the peripheral blood (66). Interestingly, Saint-Georges et al. showed that BCR stimulation also downregulated CXCR4 via protein kinase D (PKD) phosphorylation (107). Consistent with these reports, Ghobrial et al. showed that CXCR4 expression is decreased in bone marrow or lymph nodes (108). This downregulation of CXCR4 (coupled to the high expression of CD5) is therefore used to identify cells that recently emigrated from tissue into the blood circulation (105). CXCR4 activation triggers numerous intracellular pathways, including the PI3K (82), signal transducer and activator of transcription 3 (STAT3) (66), and p44/42 mitogen-activated protein kinase (MAPK) (106) pathways, leading to BTK (109), ERK (110), and AKT serine/threonine kinase 1 (AKT) (111) activation as well as calcium released (66). Interestingly, BTK is rapidly activated by CXCL12 in leukemic cells, indicating once more that the CXCL12/CXCR4 axis is interconnected with the BCR pathway (109). Similar to the CXCR4/CXCL12 axis, the CXCR5/CXCL13 axis also plays a role in CLL homing and trafficking, since CXCR5 is overexpressed on the CLL B cell surface and CXCL13 is secreted by stromal cells in B cell areas of secondary lymphoid tissues (112): FDCs produce CXCL13 which directs B lymphocytes to the “light zone” of the germinal center (5). Using the Eμ-Tcl1 mouse model of CLL, Heinig et al. demonstrated that CXCR5 depletion reduces Eμ-Tcl1 leukemogenesis, CLL proliferation and that this chemokine is indispensable for the recruitment of CLL cells into the germinal center since CXCR5-defective cells localized in the marginal zone of the B-cell follicle (10). In addition, these authors observed that lymphotoxin α and β produced by CLL B cells stimulated FDCs to produce CXCL13, suggesting that CLL/FDC reciprocal interactions leads to stromal compartment remodeling (10).

The levels of several cytokines produced by leukemic cells are dysregulated compared to those of healthy donors (113), but the basal level can also be influenced by coculture with MSCs, as demonstrated by Trimarco et al., who found that coculture induced an increase in the production of interleukin 8 (IL-8), (C-C motif) chemokine ligand 4 (CCL4), CCL11, and CXCL10 in the supernatant (114). However, based on mRNA expression, Plander et al. showed that the increases in IL-6 and IL-8 were due to MSCs (115). Other authors suggested that the major IL-6 source in a coculture system is the leukemic compartment (116). In addition, MSCs in coculture produce IL-1β, while CLL B cells produce tumor necrosis factor α (TNFα), suggesting that coculture creates an inflammatory environment (115). Interestingly, IL-8 induces prolonged survival of CLL B cells *in vitro* in an autocrine manner (117). The increase in CXCL10 was specific to CLL/MSC coculture and was not observed with normal B cells, suggesting a potential role in CLL pathophysiology (114). Moreover, CXCR3, the CXCL10 receptor, is expressed on the CLL B cell surface and mediates chemotaxis (118). CCL4 and CCL3 have also been reported to be increased in CLL B cells after coculture with nurse-like cells (NLCs) or after BCR stimulation (42). It is believed that CCL4 and CCL3 attract (C-C motif) chemokine receptor (CCR5)-positive regulatory T cells (119) or monocytes/macrophages *in vivo* in conditions that could confer survival signals to CLL B cells (42, 120–122). Using CLL coculture with the human stromal cell line HS-5, Schulz et al. similarly observed an increase in CCL2 secretion by stromal cells that was involved in the recruitment of macrophages (94). Other cytokines that can rescue primary CLL cells from apoptosis, such as IL-1α and IL-15, are also produced by MSCs after CLL contact by inducing PKC-β in stromal cells (123). IL-15 is also produced by human FDCs *in vivo* and by an FDC cell line *in vitro* (6), and has a paracrine and autocrine effect. Indeed, IL-15 is captured by IL-15Rα on the surface of FDC/HK cells and this membrane-bound form could, by transpresentation from FDCs to germinal center B cells via cell-cell contact, trigger IL-15 signaling in B cells (6) but also enhance human primary FDCs proliferation and regulate their cytokine secretion (4). In line with these observations in normal B cells, the addition of IL-15 in CLL/FDC coculture enhances CLL proliferation (10).

In secondary lymphoid organs, similarly to NLCs, FDCs produce the B cell-activating factor of tumor necrosis factor family (BAFF), an essential factor for B cell homeostasis (124, 125) but also for the survival of CLL cells (126). Endo et al. demonstrated that BAFF supports CLL B cell survival through the activation of the canonical NF-κB pathway after binding to the B-cell maturation antigen (BCMA) or the transmembrane activator and calcium modulator and cyclophilin ligand-interactor (TACI), two BAFF receptors (65).

**Figure 3:**
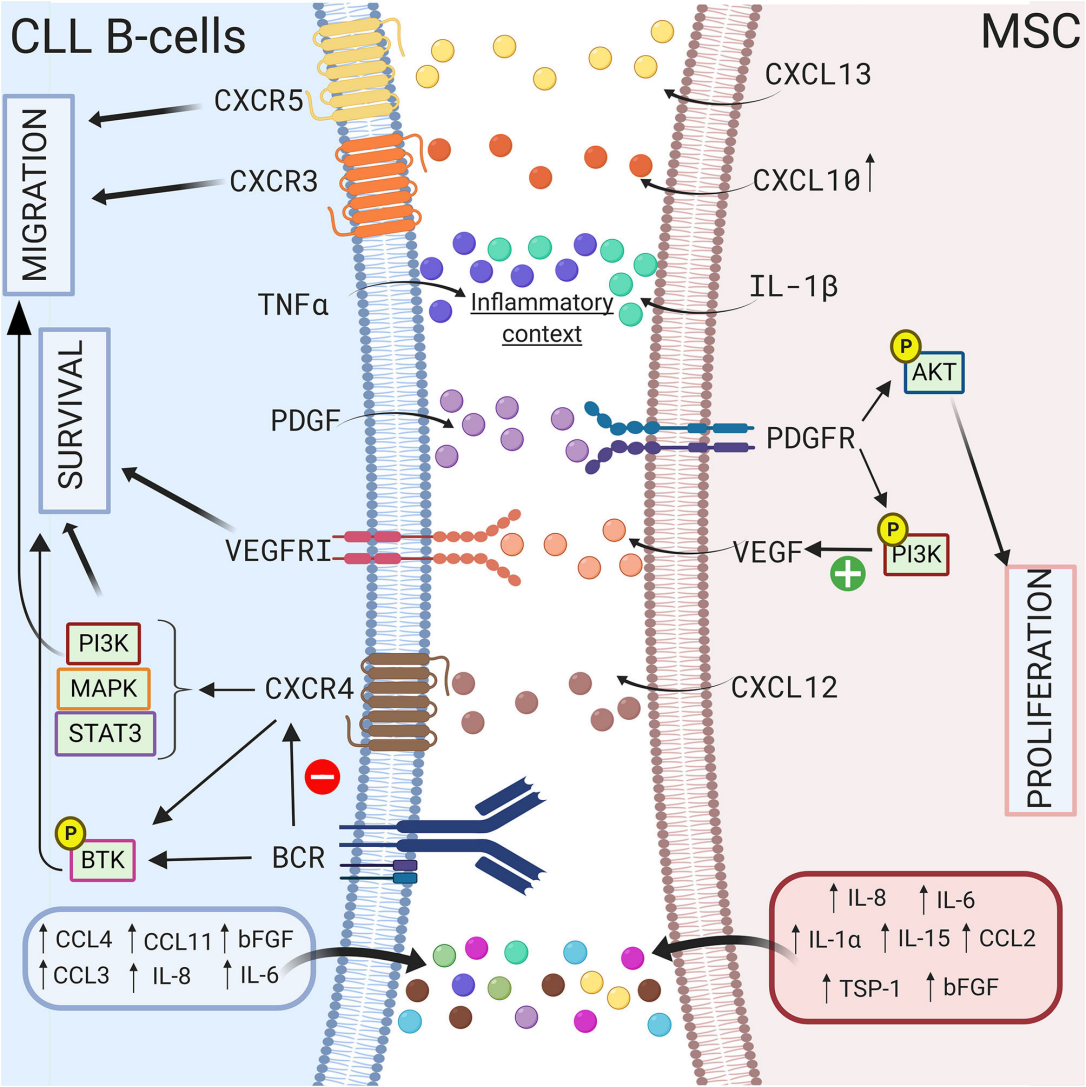
Crosstalk between CLL B-cells and MSCs via soluble factors. CLL B-cells interact with MSCs by several soluble factors including cytokines (115, 117), chemokines (42, 114), and growth factors (69, 127, 128). MSCs can produce CXCL10, CXCL13, CXCL12 that binds to their respective receptor on CLL B-cells CXCR3 (118), CXCR5 (112), CXCR4 (66, 69, 75). The triggering of CXCR4/CXCL12 axis lead to the activation of several pathways including PI3K (82), MAPK (106), or STAT3 (66) leading to the survival and the migration of the leukemic cells. Interestingly, BCR stimulation induces the downregulation of CXCR4 (107), the activation of BTK (109), and the increased secretion of some cytokines. MSCs in coculture also produce IL-1β while CLL B-cells produce TNFα suggesting that coculture creates an inflammatory context (115). Figure created with BioRender.com.

Growth factors also sustain CLL B cell survival in a coculture model. Gehrke et al. observed that VEGF produced by HS-5 stromal cells but not CLL B cells is essential for their coculture-mediated survival (127). Our group also suggested that compared to healthy MSCs, the increased secretion of TGFβ1 by CLL-derived MSCs play a crucial pathogenic role in CLL (50). In addition, Kay et al. observed an increase in VEGF, thrombospondin-1 (TSP-1) and basic fibroblast growth factor (bFGF) in the supernatant of a coculture system of primary CLL B cells and CLL patient-derived MSCs (69). These authors suggested that this increased secretion was due to MSCs (69). In 2009, Ding et al. observed activation of the AKT pathway in MSCs in coculture with leukemic cells, supporting the existence of bidirectional interactions (68). One year later, the same authors demonstrated that the platelet-derived growth factor (PDGF) produced by CLL B cells is responsible for this activation and induces VEGF production in MSCs in a PI3K-dependent manner (128). A schematic representation of the different interactions between CLL B cells and MSCs via the production of soluble factors is shown in Figure 3.

## Extracellular Vesicles: A New Way of Crosstalk

In recent years, a new method of intercellular communication via the exchange of extracellular vesicles (EVs) has been described. Observed 50 years ago as “platelet dust” (129), EVs were long considered cellular debris, but today, they are known to play important roles in several pathophysiological processes, including immune responses, tissue regeneration, blood coagulation (130), and crosstalk between normal/cancer cells (131). EVs can be divided into 2 different groups based on their origin: exosomes and microvesicles (also known as ectosomes). Exosomes are released from multivesicular bodies (late endosomes) at the plasma membrane and generally have a size ranging from 30 to 150 nm, while microvesicles result directly from plasma membrane budding and pinching and have a size between 100 nm and 1 μm (132, 133). EVs can shuttle and transfer their content from one cell to another. Several reports have described that EVs carry DNA fragments, different species of RNA (mRNA, Y RNA, and microRNA), proteins, peptides or lipids (134–137).

To date, few reports have described the role of EVs in CLL B cells and MSC communication. Crompot et al. were the first to highlight the impact of bone marrow MSC EVs on CLL B cells *in vitro* (138). These authors showed that MSC-derived EVs are rapidly incorporated in CLL B cells and that they increase CLL cell migration, suggesting that these EVs could give CLL cells survival advantages *in vivo*. In addition, MSC-derived EVs protect leukemic cells from spontaneous and drug-induced apoptosis, as well as induce gene expression modifications. Overlap of gene signatures induced by EVs with other microenvironmental stimuli [such as BCR stimulation (139) or NLC coculture (42)] suggested that a substantial part of cell-to-cell communication is mediated by EVs. Finally, several (but not all) effects of MSC-derived EVs mimic BCR stimulation, which has been described as crucial in CLL B cell survival (139, 140).

**Figure 4:**
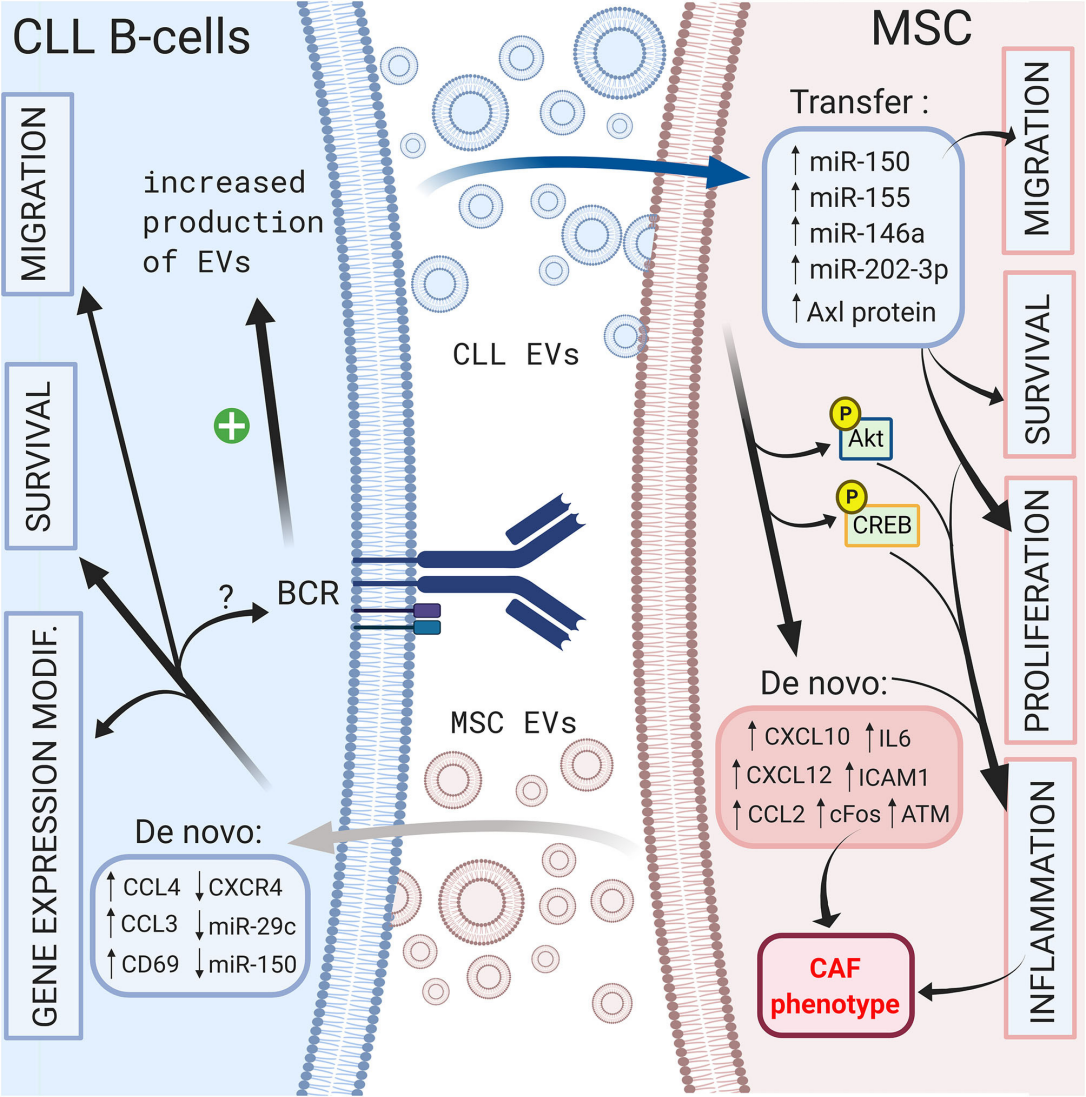
Crosstalk between CLL B-cells and MSCs via extracellular vesicles. Bi-directional communication exists between CLL B-cells and MSCs via the production of extracellular vesicles by both cell types. MSC EVs increase the migration, the survival of CLL B-cells and change their gene expression profile (138). CLL B-cells derived EVs can transfer microRNA (135, 144, 145) or protein (143) leading to the migration, the survival and the proliferation of MSCs (135). In addition, they induce an inflammatory phenotype in stromal cells resulting into a cancer-associated fibroblast (CAF)-phenotype (135). Interestingly BCR stimulation increases the production of CLL EVs (145). Figure created with BioRender.com.

On the other hand, CLL B cells also release EVs that can modulate their microenvironment. Paggetti et al. demonstrated that exosomes can be taken up by stromal cells and transfer microRNA-150 (29), microRNA-155 (141) and microRNA-146a (142), which have been described in CLL B cells (135). Moreover, leukemic exosomes induce an inflammatory phenotype in stromal cells by increasing AKT and cyclic AMP response element binding protein (CREB) phosphorylation via the NF-κB pathway, resulting in a cancer-associated fibroblast (CAF)-phenotype (135). Interestingly, this phenomenon is coupled to the increase in VEGF, CXCL10, CCL2, IL6, intercellular adhesion molecule 1 (ICAM1) or CXCL12, which are important molecules involved in the homing of CLL cells that were previously discussed (135, 143). Ghosh et al. reported that CLL microvesicles carry Axl protein that could be transferred to MSCs, leading to an increase in AKT phosphorylation (143). Farahani et al. showed that CLL exosomes encapsulate an abundant amount of microRNA-202-3p that, once integrated in stromal HS-5 cell lines, enhanced their proliferation and decreased apoptosis by inducing the expression of genes such as c-fos and ataxia telangiectasia mutated (ATM) (144). Interestingly, several authors observed an enrichment in specific microRNAs in exosomes compared to that of the cell compartment (135, 144, 145). As explained previously, BCR stimulation is crucial for CLL B cell survival, and multiple microenvironmental stimuli, such as MSC or NLC coculture, could trigger BCR signaling. In this context, Yeh et al. observed that BCR stimulation increases exosome production by CLL B cells but also modifies their microRNA-150 and microRNA-155 content (145). These data suggest that different microenvironmental stimuli could be amplified via EVs. A schematic representation of the influence of EVs in CLL B cell/MSC crosstalk is shown in Figure 4.

## Targeting CLL/MSC Crosstalk

As explained previously, CLL B cells migrate from the peripheral blood to tissues in response to different chemokines. When leukemic cells reach the stromal microenvironment, they enter a protective niche against drug-induced apoptosis. Therefore, mobilizing these cells out of these niches to increase their chemosensitivity has been a proposed strategy. The first studies tried to inhibit CLL/MSC crosstalk by acting extracellularly on the CXCR4/CXCL12 axis. We showed that AMD3100 (also known as plerixafor), a bicyclam molecule and specific antagonist of the CXCR4 receptor (146), prevents the binding of CXCL12 and results in a decrease in pseudoemperipolesis and an increase in chemosensitivity to different drugs (71). Recently, a clinical trial combining plerixafor and rituximab (an anti-CD20 antibody) confirmed an increase in cell mobilization in peripheral blood but an overall response rate of 38% (147). Other authors tried to target this axis using a CXCR4 antibody (148) or by decreasing the expression of CXCR4 using a histone deacetylase inhibitor (70, 149). Another proposed strategy was to inhibit the ligand CXCL12 and not the receptor. NOX-A12, an RNA oligonucleotide in the L-configuration that binds and neutralizes CXCL12, has been shown to decrease CLL B cell migration and increase chemosensitivity but surprisingly increases pseudoemperipolesis (150). All authors agree that inhibition of the CXCR4/CXCL12 axis is only an adjuvant and therefore should always be coupled with a cytotoxic drug.

**Figure 5:**
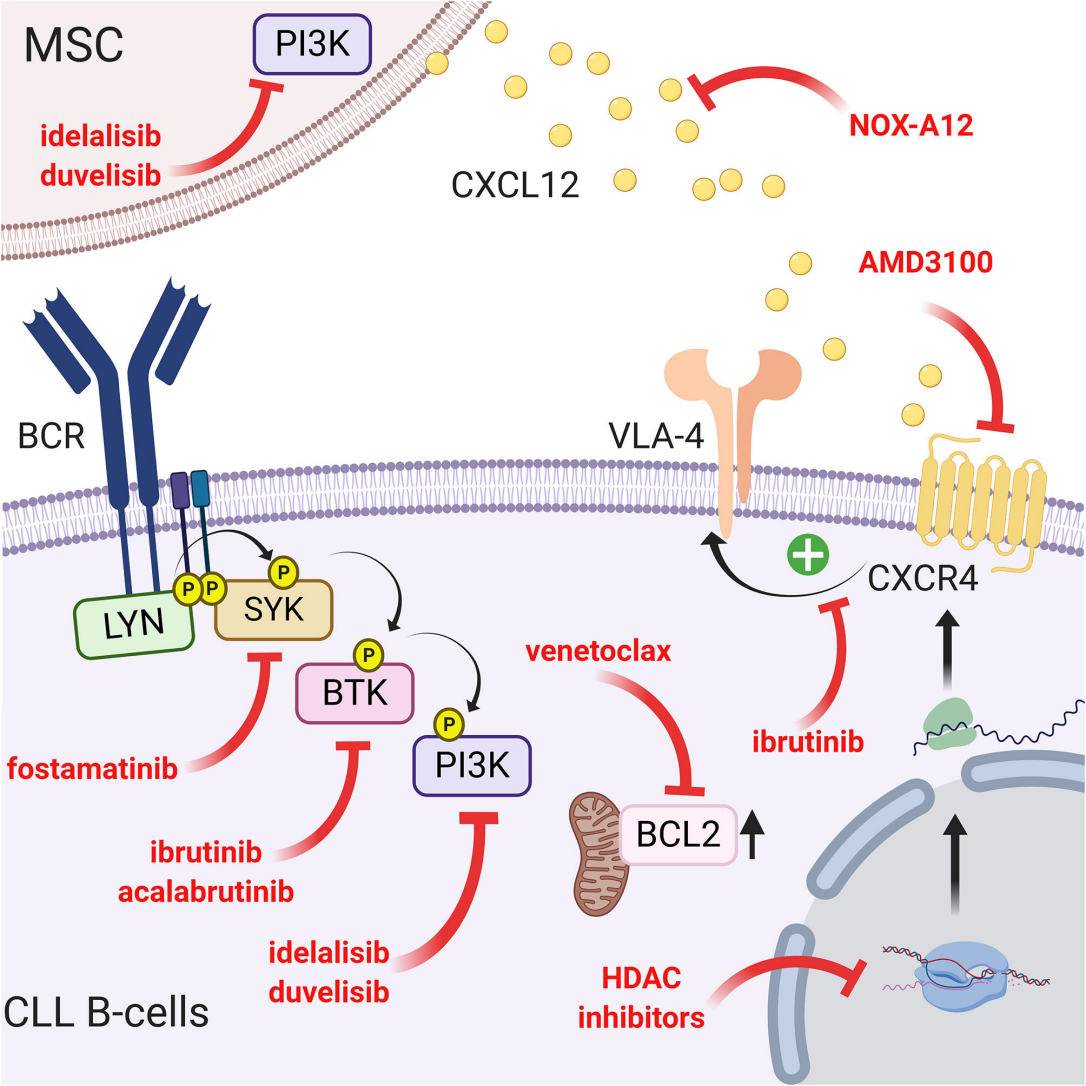
Targeting the CLL/MSC crosstalk using CXCR4/CXCL12 and BCR signaling inhibitors. Several therapeutic strategies have been proposed to inhibit CLL/MSC interactions. (a) blocking of CXCR4 using AMD3100 (71) or using a CXCR4 antibody (148); (b) blocking CXCL12 using NOX-A12 (150); (c) CXCR4/CXCL12 and BCR pathways are interconnected, another way to interfere is to target BCR pathway using different inhibitors: ibrutinib that inhibits BTK and downstream events such as MAPK, PI3K, or NF-κB activation (151) but also reduces VLA-4 activation induced by CXCL12 binding (109) and cell adhesion (79). Acalabrutinib (155) that similarly acts on the migration, the homing and the mobilization of leukemic cells in the circulation. Idelalisib or duvelisib also reduced the ability of stromal cells to support CLL migration and adhesion (161); (d) finally, targeting the over-expression of BCL2 partially induced by the microenvironment has also been proposed (163, 164). Figure created with BioRender.com.

Since the CXCR4/CXCL12 and BCR pathways are interconnected, another way to interfere with the migration and homing of leukemic cells into a protective microenvironment is to target the intracellular pathways using specific drugs. One of the more potent and recently discovered drugs is ibrutinib (previously called PCI-32765), a Bruton's tyrosine kinase inhibitor (37). This small molecule acts by covalently binding cysteine 481 in the active site of BTK and consequently inhibits downstream events such as MAPK, PI3K, or NF-κB activation (151). This inhibition therefore results in a drastic reduction in migration and adhesion of CLL B cells in the lymphoid tissue (particularly the lymph node) and their mobilization in peripheral blood (152, 153). In this context, Tissino et al. reported a relationship between ibrutinib exposure and impaired CLL cell adhesion on VCAM-1 substrates *in vitro* and a progressive reduction of constitutive VLA-4 activation during *in vivo* ibrutinib treatment (79). However, decrease of VLA-4 activation by ibrutinib is still under debate since BTK inhibition could be bypassed by triggering the CXCR4/CXCL12 axis (154) or by an exogenous BCR stimulation in a BTK-independent manner involving PI3K (79). In contrast, other authors observed that BTK inhibition prevents CXCL12-induced triggering of LFA-1 and VLA-4 integrins (109). In addition, ibrutinib also reduces the surface level of CXCR4 by inhibiting cycling from and to the membrane (153). Not surprisingly, other drugs targeting BCR pathways, such as a more specific BTK inhibitor (acalabrutinib; previously named APC-196) (155), SYK inhibitor (fostamatinib) (156) or PI3K inhibitor (idelalisib, duvelisib) (157, 158), will have very similar effects on migration, homing and mobilization of leukemic cells in the circulation by inhibiting chemotaxis in response to CXCL12 and CXCL13 and reducing adhesion to VCAM1 and fibronectin (159, 160). Complementarily, idelalisib or duvelisib also significantly reduced the ability of stromal cells to support CLL migration and adhesion (161). Another way to disrupt CLL/MSC crosstalk and overcoming drug resistance in CLL patients is to directly target PKC-β signaling pathway in MSC: indeed, Park et al. showed that small-molecule PKC-β inhibitors antagonize prosurvival signals from stromal cells and sensitize tumor cells to targeted and non-targeted chemotherapy, resulting in enhanced cytotoxicity (162). In addition, they also showed that stromal PKC-β controls the expression of adhesion and matrix proteins, required for activation of PI3Ks and ERK-mediated stabilization of BCL-XL in tumor cells (162). Microenvironment stimuli provided during CLL/MSC coculture lead to the increase of BCL2 through Notch-1, Notch-2, Notch-4 signaling (97). This could partially explain the high level of BCL2 expression in CLL, real hallmark of leukemic cells. Therefore, targeting BCL2 overexpression has been proposed using venetoclax (or ABT-199), an efficient and selective small-molecule inhibitor for BCL2 (163, 164). A schematic representation of the different targeting strategies to overcome the protection of the microenvironment is shown in Figure 5.

## The Microenvironment and Prognostic Factors

For almost 20 years, a plethora of prognostic markers have been described to stratify CLL patients (165). Many of these markers are linked to the capacities of CLL B cells to interact with their microenvironment (166). The gold standard prognostic marker is the mutation status of IgHV (167). Coscia et al. observed that cells obtained from IgHV normal patients are more prone than those with mutated IgHV cells to undergo spontaneous apoptosis *in vitro*, suggesting that unmutated cells are more dependent on survival stimuli from the microenvironment (168). In addition, patients with unmutated IgHV have been shown to be more responsive to external stimuli such as the BCR stimulation by downregulating CXCR4 and CD62L (169, 170). These *in vitro* results suggest that in unmutated IgHV CLL patients, leukemic cells would be more likely to respond to MSC stimuli and be more “BCR activable” in an *in vivo* situation leading to cell survival. Several surrogate markers for IgHV mutation status have also been described (165). Of these, ZAP70 expression has been strongly associated with prognosis (22, 171). Interestingly, numerous authors have shown that ZAP70+ CLL B cells have better migratory capacities and that they are characterized by a gene signature linked to migration, homing or CXCR4/CXCL12 pathways (166, 172, 173). Again, based on these *in vitro* migration capacities, we could speculate that in ZAP70+ patients, cells could have more opportunities to interact with MSCs but also other protecting cells in the *in vivo* bone marrow and lymph node microenvironment. We also observed CXCR4 downregulation on CLL B cells from patients with a poor prognosis when they were co-cultured with MSCs, as well as an increase in CD69 surface expression (166). The CXCR4 level was correlated with leukocyte count (174), and when combined with CXCR3 expression, it has been proposed as a prognostic marker to predict the time to first treatment (TTFT) (175, 176). The lower expression of CXCR4 has also been observed *in vivo* on CLL cells isolated from bone marrow or lymph node compared to peripheral blood (177). Therefore, it is believed that CXCR4 downregulation in the tissues allows a recirculation of leukemic cells in peripheral blood creating by this way their shuttling between the different body compartments (18, 66, 105). Based on this hypothesis, CXCR4^high^ CLL cells would be more efficiently attracted to bone marrow MSCs *in vivo* and, after contact, will downregulate CXCR4. The increase in CD69 on leukemic cells in coculture with MSCs (68, 166) or after treatment with MCS-derived EVs (138) is also linked to prognosis, since CD69 positivity is associated with shorter progression-free survival (PFS) and overall survival (OS) (26). The higher expression of CD69 has also been observed *in vivo* on CLL cells isolated from bone marrow or lymph node compared to peripheral blood (147) and is the reflection of the CLL activation status. Another example is the high expression of CD38, which has been described as an independent prognostic marker in CLL (24, 178) and is upregulated on CLL B cells after 2 weeks in coculture with stromal cells (68). However, it should be noted that this marker can change during disease course, suggesting that MSC/CLL *in vivo* interactions can also vary during disease. As described above, leukemic cells can also receive BCR stimulation from the microenvironment that induces dramatic changes in their gene expression and secretion (139). Pallasch et al. demonstrated that lipoprotein lipase (LPL) is increased in CLL B cells by BCR stimulation (179). LPL has been described as a strong prognostic marker (171, 180, 181). The release of CCL3 and CCL4 after coculture with NLCs (42) or MSCs (114) or after treatment with MCS-derived EVs (138) is another example showing that the consequences of microenvironmental interactions could be used as prognostic factors, since CCL3 and CCL4 levels in the plasma of CLL patients are associated with the time from diagnosis to initial therapy (121). Herishanu et al. observed that CLL3 and CCL4 expression is increased in CLL cells from bone marrow and even more from lymph node (177) suggesting that CLL/MSC and CLL/NLC interaction probably also occurs *in vivo*. A similar conclusion could be drawn for the increased secretion of IL-8 (114, 115), PDGF (128), and VEGF (127) by MSCs in co-culture and the association of elevated plasma levels of these factors with high-risk factors and more advanced stage in CLL patients (128, 182). The decrease in microRNA-29c after BCR stimulation (138) could also explain its prognostic power (27). CLL B cells in coculture with MSCs significantly upregulate the expression of CD49d (183), which is also a very strong prognostic marker (184–186). Taken together, these data suggest that several prognostic markers are the direct consequence of leukemic cell interactions with the microenvironment while others which do not change along time (such as unmutated IgHV) define patients whose cells are more able to respond to microenvironmental stimuli *in vivo*. Table 1 summarizes the different prognostic markers, their prognostic power in CLL and their link with the microenvironment.

**Table 1:** Summary of CLL prognostic markers linked to microenvironment interactions.

**Prognostic factor**	**Correlation**	**References**	**Poor prognosis**	**Link with microenvironment**	**References**
IgHV	TFS, OS	(20)	Unmutated (UM)	UM are more prone to apoptosis, more dependent to microenvironment stimulus	(168)
		(24)		UM are associated with an ability to respond to BCR	(169)
				Stimulation by downregulation CXCR4 and CD62L	(170)
ZAP-70	TFS, OS	(22)	High expression	ZAP70+ CLL B-cells have better migration capacities	(172, 173)
		(171)		Gene signature linked to migration, homing or CXCR4/CXCL12 pathways	(166)
CXCR4/ CXCR3	Leucocyte counts, TTFT	(174–176)	Low expression	Decrease in coculture, after BCR stimulation	(137, 166)
CD69	PFS, OS	(26)	Positive	Increase on CLL cells in coculture with MSCs	(68, 166)
				Increase on CLL cells after treatment with MSC EVs	(138)
CD38	TFS, OS	(24, 178)	Positive	Increase on CLL B-cells after 2 weeks in coculture with stromal cells	(68)
LPL	TFS, OS	(171, 180, 181)	High expression	Increase in CLL B-cells after a BCR stimulation	(179)
CCL3/ CCL4	TFS	(121)	High plasma level	Increase after coculture with NLCs	(42)
				Increase after coculture with MSCs	(114)
				Increase after treatment with MCS-derived EVs	(138)
IL-8	Other markers, OS	(182)	High plasma level	Increase in the supernatant of CLL/MSC coculture	(114, 115)
PDGF	ZAP-70, CD38, need of therapy	(128)	High plasma level	PRGF receptor were selectively activated in MSCs by CLL conditioned medium PDGF is were detected in CLL conditioned medium	(128)
VEGF	ZAP-70, CD38, need of therapy	(128)	High plasma level	VEGF is detected in CLL conditioned medium PDGF induced MSC VEGF production	(127)
miR-29c	TFS, OS	(27)	Low expression	Decrease after BCR stimulation	(138)
CD49d	TFS, OS	(184–186)	Positive	Increase on CLL B-cells in coculture with MSCs	(183)

## Conclusions

Over the last two decades, many reports have demonstrated the different ways CLL B cells and stromal cells in the bone marrow and lymph node communicate. These cell interactions are bidirectional, inducing many changes in both cell types: dysregulation of adhesion molecules, abnormal secretion of cytokines, chemokines and growth factors and modification of normal trafficking and homing. CLL cells are able to crosstalk with close surrounding cells by direct cell-to-cell contact and can communicate with distant cells via the production of extracellular vesicles. CLL cells can modify healthy cells in different ways, altering them from their physiological functions. All these different interactions make it difficult to study this topic exhaustively, but recent studies have highlighted crucial and targetable pathways. Targeting BCR signaling has been shown to mobilize leukemic cells out of their protective microenvironment. Some new small molecules have already demonstrated their efficacy in CLL patients, improving their overall survival. Our knowledge of how leukemic cells are able to interact also brings out a plethora of prognostic markers that are only a reflection of how efficient this crosstalk is. Despite some indirect data (such as serum level of some cytokines or gene expression from CLL cells isolated from different body compartments), the vast majority of the data we have access to today about CLL/MSC interactions derive from *ex vivo* studies. The *ex vivo* expansion of MSCs requiring multiple passages, the use of stromal cell lines, the isolation of CLL cells from patient blood or the use of leukemic cell lines could not reflect all the aspects of the *in vivo* situation. Therefore, further studies are needed to extensively understand the “true” *in vivo* CLL/MSC biology. However, understanding CLL/microenvironment communication has already helped us discover new treatment strategies, but further functional characterizations will open new ways to avoid patient relapse in the future.

## Author Contributions

ND and BS: conception, design, and writing of the manuscript. ND, EC, NM, DB, LL, and BS: review, and/or revision of the manuscript. All authors contributed to the article and approved the submitted version.

## Conflict of Interest

The authors declare that the research was conducted in the absence of any commercial or financial relationships that could be construed as a potential conflict of interest.

## Abbreviations

AKT: AKT serine/threonine kinase 1
ATM: ataxia telangiectasia mutated
BAFF:B cell-activating factor of tumor necrosis factor family
BCL2: B-cell lymphoma 2
BCL-XL: B-cell lymphoma-extra-large
BCMA: B-cell maturation antigen
BCR: B-cell receptor
bFGF: basic fibroblast growth factor
BTK: Bruton's tyrosine kinase
CAF: cancer-associated fibroblast
CCL: (C-C motif) chemokine ligand
CCR: (C-C motif) chemokine receptor
CLL: chronic lymphocytic leukemia
CR: complement receptor
CREB: cyclic AMP response element binding protein
CXCL: C-X-C motif chemokine ligand
CXCR: C-X-C motif chemokine receptor
Del: deletion
ERK: extracellular regulated kinases
EVs: extracellular vesicles
FDC: follicular dendritic cells
H3K27me3: trimethylation of lysine 27 on histone H3 protein subunit
IC: immune complex
ICAM1: intercellular adhesion molecule 1
Ig: immunoglobulin
IgHV: immunoglobulin heavy variable region chain
IL: interleukin
ISCT: International Society for Cellular Therapy
ITAM: immunoreceptor tyrosine-based activation motifs
ITGB: integrin beta
JAK: Janus kinase
LFA-1: lymphocyte function-associated antigen 1
LPL: lipoprotein lipase
LYN: Lck/Yes novel tyrosine kinase
MAPK: mitogen-activated protein kinase
Mcl-1: myeloid leukemia cell differentiation protein 1
miR: microRNA
MSC: mesenchymal stromal cells
NFκB: nuclear factor kappa B
NLC: nurse-like cells
Notch2: neurogenic locus notch homolog protein 2
OS: overall survival
PDGF: platelet-derived growth factor
PFS: progression free survival
PI3K: phosphoinositide 3-kinase
PKC: protein kinase C
PKD: protein kinase D
SDF1: stromal cell derived factor 1
STAT3: signal transducer and activator of transcription 3
SYK: spleen tyrosine kinase
TACI: transmembrane activator and calcium modulator and cyclophilin ligand-interactor
TGFβ1: transforming growth factor β1
TNFα: tumor necrosis factor α
TP53: tumor protein 53
TSP-1: thrombonspondin-1
TTFT: predict time to first treatment
VCAM-1: the vascular cell adhesion molecule 1
VEGF: vascular endothelial growth factor
VLA-4: very late antigen-4
Wnt: wingless integration site
ZAP70: zeta-associated protein 70.
